# Precipitation modulates the net effect of solar radiation on litter decomposition and CO_2_ emission - a meta-analysis

**DOI:** 10.3389/fpls.2023.1200155

**Published:** 2023-07-05

**Authors:** YaLan Liu, Lei Li, ShiQi Wang, Xiangyi Li

**Affiliations:** ^1^ State Key Laboratory of Desert and Oasis Ecology, Xinjiang Institute of Ecology and Geography, Chinese Academy of Sciences, Urumqi, China; ^2^ Xinjiang Key Laboratory of Desert Plant Roots Ecology and Vegetation Restoration, Xinjiang Institute of Ecology and Geography, Chinese Academy of Sciences, Urumqi, China; ^3^ Cele National Station of Observation and Research for Desert-Grassland Ecosystems, Cele, China; ^4^ University of Chinese Academy of Sciences, Beijing, China

**Keywords:** meta-analysis, precipitation, litter, photodegradation, microbial decomposition

## Abstract

**Introduction:**

Solar radiation plays a crucial role in the decomposition of litter and the cycling of nutrients. Previous studies have investigated that the net effect of solar radiation on litter decomposition depends on the balance of its facilitative and inhibitory effects on microbial activity; however, a gap in understanding the mechanism by which precipitation affects the net effect of solar radiation and the mechanism of litter decomposition on a global scale was observed.

**Methods:**

In addressing this gap, a comprehensive meta-analysis of 351 data points from 37 published studies was conducted to estimate the sole radiation effect and interactive effect of solar radiation and precipitation on a global scale, as well as how they vary at different precipitation levels. In addition, the importance of influential factors regulating the net effect of solar radiation on litter decomposition was assessed to identify the key drivers of the response of mass loss to solar radiation at different precipitation levels.

**Results:**

Our findings indicated that solar radiation largely regulates litter decomposition, and the direction and magnitude are potentially dependent on the precipitation regime. In addition, solar radiation significantly increased mass loss and decreased the nutrient remaining. Furthermore, the effects of solar radiation on mass loss, C remaining, and N remaining were found to be similar among areas with precipitation levels below 200 and above 800 mm and greater than in areas with precipitation levels between 200-400 mm and 400-800 mm. The effect of solar radiation on CO_2_ emissions varied from 13.97% when precipitation was below 200 mm to −0.707% when precipitation was between 200 and 400 mm.

**Conclusion:**

Climatic factors determine the response ratio of mass loss to solar radiation in arid lands, whereas the initial litter characteristics have a great influence on the response of mass loss to solar radiation in ecosystems that are not moisture limited. The effect of precipitation on the photodegradation mechanism of litter was primarily achieved by influencing the decomposition of lignin, and the main effect of solar radiation on litter decomposition will shift from the positive effect of “photopriming” to the negative effect of “microbial inhibition” with the increase of precipitation. Our findings can provide a comprehensive understanding of litter decomposition patterns on a global scale, and our results showed that CO_2_ emissions from photodegradation will be lessened by precipitation, which is important in predicting CO_2_ emission and separating sources of CO_2_ under future increasing precipitation scenarios, particularly in arid lands.

## 1 Introduction

Litter decomposition is a crucial biogeochemical process that plays a vital role in the cycling of carbon and other nutrients in ecosystems (Swift et al., 1979; Wang et al., 2021). Various factors, such as microorganisms, local climate, and litter quality, are considered as primary determinants of litter decomposition (Coûteaux et al., 1995). Empirical models that incorporate these three factors have been developed to estimate litter decomposition in diverse ecosystems (Bonan et al., 2013). However, these models often underestimate the decomposition rate in semiarid and arid lands, and they cannot fully account for the variation of decomposition among different terrestrial ecosystems (Bonan et al., 2013). These findings indicate that additional factors may influence litter decomposition. Therefore, exploring more comprehensive mechanisms that affect litter decomposition and carbon cycling is essential to predict changes in carbon and nutrient dynamics in the face of climate change.

In the past few decades, scientists have increasingly recognized the impact of solar radiation on litter decomposition, particularly in arid lands where photodegradation can account for approximately 26% of mass loss (Adair et al., 2017). On the one hand, solar radiation can directly affect recalcitrant organic matter such as lignin, breaking it down into smaller, more easily decomposable organic materials and directly producing greenhouse gases such as CO_2_ (Pancotto et al., 2003; Austin and Vivanco, 2006; Gallo et al., 2006; King et al., 2012; Austin et al., 2016). In addition, these smaller organic materials can indirectly enhance microbial decomposition by promoting microbial and enzymatic activities (King et al., 2012; Austin et al., 2016; Berenstecher et al., 2020). On the other hand, solar radiation can also inhibit microbial decomposition because microbes can absorb photons, which caused DNA damage (Johnson, 2003; Pancotto et al., 2003). Therefore, the net effect of solar radiation on litter decomposition depends on the balance between its facilitative and inhibitory effects on microbial activity (Johnson, 2003; Robson et al., 2005; Huang and Li, 2017; Lin et al., 2017; Wang et al., 2021).

Precipitation is a significant factor regulating the response of litter decomposition to solar radiation (Smith et al., 2010; Huang and Li, 2017). In semi-arid and arid regions, the positive effect of solar radiation on litter decomposition can offset the negative effect on microbes, and the net effect of solar radiation on litter decomposition is generally positively related to the amount of precipitation (Wang et al., 2015; Gliksman et al., 2017; Huang and Li, 2017; Pieristè et al., 2020). An increase in precipitation directly enhances litter leaching, facilitates nutrient dissolution, and produces more dissolved organic carbon (DOC), which can be easily degraded by solar radiation (Pancotto et al., 2003; Smith et al., 2010). However, the relative importance of photodegradation in the decomposition of litter remains unclear as some field studies have shown that precipitation can simultaneously increase photodegradation and microbial decomposition, and no assessment has been made of whether the improvement in photodegradation exceeds that of microbial decomposition (Smith et al., 2010; Huang and Li, 2017).

Similarly, the net effect of solar radiation on litter decay in relatively humid ecosystems and the mechanism by which it changes with increasing precipitation remain unclear with conflicting findings reported in various studies (Smith et al., 2010; Evans et al., 2020). These conflicts may be due to variations in litter traits, with some plants developing more protective structures, such as lignin, which make them more sensitive to solar radiation (Gehrke et al., 1995; Moody et al., 2001; Austin and Vivanco, 2006; Lin et al., 2017; Wang et al., 2021). Other factors that can reduce the contribution of litter photodegradation include periodic snow cover and a dense plant canopy, which can decrease cumulative irradiance (Egidi et al., 2023; Smith et al., 2010; Evans et al., 2020). In addition, experiments in relatively humid conditions have shown that the net effect of radiation shifts from “photopriming” to biological suppression with increasing precipitation (Moody et al., 2001; Pancotto et al., 2003; Smith et al., 2010). As future extreme precipitation is expected to increase, particularly in 40% of the global land area that is dryland (Reynolds et al., 2007; Yao et al., 2020), the mechanism of litter decomposition in global drylands will undergo fundamental changes. Therefore, understanding the mechanism of nutrient loss in the litter, particularly in arid ecosystems, is crucial for effectively predicting and managing global carbon and nutrient cycles.

In investigating our research questions, a comprehensive meta-analysis of photodegradation studies was conducted to examine the interactive effects of solar radiation and precipitation on litter decomposition on a global scale. Moreover, the impact of solar radiation on litter decomposition was evaluated across various mean annual precipitation (MAP). The following specific questions were addressed: (1) How does the effect of radiation on litter decomposition vary with different precipitation levels? (2) Whether do the key drivers of the response of mass loss to solar radiation in different precipitation levels change? (3) Whether and how does the mechanism of photodegradation vary with different precipitation levels?

## 2 Materials and methods

### 2.1 Meta-analysis

**Figure 1:**
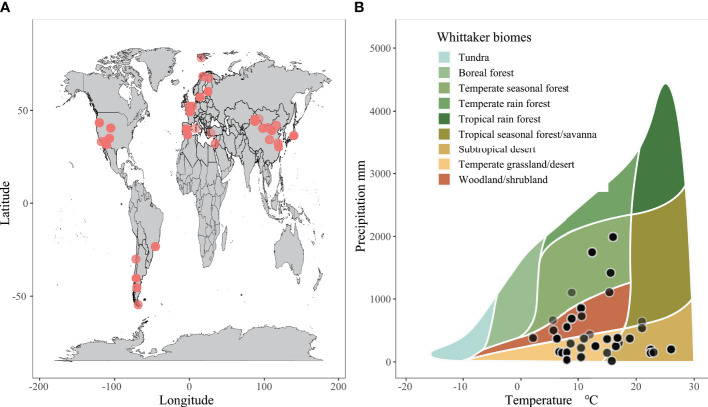
Global map of the study sites distribution **(A)**, and local climate and biomes **(B)** in this meta-analysis.

Our dataset for meta-analysis was obtained by extracting 351 data points from 37 articles found in the “Web of Science” database (**Figure 1**; **Table S1**). These articles were published between 1995 and 2022, which contained keywords such as “UV”, “Ultraviolet radiation”, “radiation”, “light”, “irradiance”, “solar”, “photodegradation”, “decomposition”, “decay”, and “degradation”. We ensured that the selection process was systematic to avoid publication bias and that the data met the following criteria: (1) experiments involving litter collections were conducted on a soil surface in natural terrestrial systems, excluding studies in lab and aquatic systems; (2) the overall litter decomposition characteristics (i.e., K value and mass loss), litter quality traits (i.e., C, N, C:N, lignin, or dissolved organic carbon (DOC)), or microbial growth characteristics (i.e., microbial biomass carbon (MBC) and CO_2_ emission) were analyzed during the experiment; (3) control and treatment experiments were consistently maintained under the same abiotic and biotic conditions; (4) the litters were exposed to a minimum of two levels of UV radiation, with the treatment group being exposed to ambient UV and the control group being subjected to conditions in which UV was blocked or reduced; (5) only the latest results were collected for experimental observations spanning multiple years; (6) if an article included multiple sites and species, each species and site were treated as an independent study.

In estimating the interactive effect of precipitation and radiation on litter decomposition, the observation was divided into two types, namely, “Radiation” and “Combine”. “Radiation” studies included photodegradation experiments that did not receive extra water addition and included 351 data points. Meanwhile, “Combine” studies included photodegradation experiments that received extra water addition artificially and included 15 data points. The interactive effect of radiation and precipitation was obtained by comparing it to the treatment without radiation and water addition. However, given the insufficient data, the interactive effect between radiation and precipitation was not explored in depth. Therefore, the studies were categorized into the following four groups based on precipitation amounts, characteristics of precipitation distribution in the data, and precipitation characteristics of Koppen’s climatic province (Beck et al., 2018): precipitation< 200 mm, 200–400 mm, 400–800 mm, and >800 mm, corresponding to arid, semiarid, subhumid, and humid lands. This technique allowed us to accurately estimate the interactive effect of precipitation and solar radiation on litter decomposition.

We extracted local site information, including latitude, longitude, mean annual temperature (MAT), MAP, experimental duration, and litter traits such as initial C, N, lignin, hemicellulose, cellulose, and DOC concentration, from the articles. Graphical data were obtained using Web Plot Digitizer 4.2. In evaluating the relationship between soil respiration and photodegradation, soil respiration data were retrieved from the Soil Respiration Database (SRDB V5) (Jian et al., 2021), which roughly corresponded to the geographic coordinates of the data points in the meta-analysis.

### 2.2 Data analysis

The meta-analysis was conducted using Metawin software. The impact of solar radiation alone and the interactive effect between solar radiation and precipitation on litter decomposition were evaluated through log response ratio (LnRR) as Hedges et al. (1999):


$$\text{LnRR}=\text{Ln}\left(\frac{Xt}{Xc}\right)=\text{Ln}\left(Xt\right)-\text{Ln}\left(Xc\right)$$

and a variance:


$$v=\frac{{\left(St\right)}^{2}}{Nt{\left(Xt\right)}^{2}}+\frac{{\left(Sc\right)}^{2}}{Nc{\left(Xc\right)}^{2}}$$

where *Xt* and *Xc* are the mean values of each variable in treatment and in the control, *St* and *Sc* are the standard deviations, and *Nt* and *Nc* are sample size. The variance in mean LnRR was computed by 95% confidence intervals.


W=(Nt∗Nc)/(Nt+Nc)

where W is the weighting factor for each LnRR datapoint. To assess the overall weighted response ratio (LnRR++) of litter decomposition to precipitation, we employed a random model to computed it.


$$\text{LnRR}++=\left(\sum_{i}{\text{LnRR}}_{\text{i}}*W_{i}\right)/(\sum_{i}W_{i})$$

The variance in mean lnRR++ was computed by 95% confidence intervals (CIs), which were produced by bootstrapping function. Mean lnRR++ and its 95% confidence intervals (CIs) were used to calculate effect sizes:


$$\text{Effect size}\left(\%\right)=({\text{e}}^{\text{LnRR}++}-1)*100\%$$

The Q test was performed to evaluate between-group heterogeneity and the subcategories, including precipitation levels (<200 mm, 200–400 mm, 400–800 mm, and >800 mm), ecosystem types (forest, shrubland, and grassland), species (tree, shrub, and herb), and experimental duration (<1 year, 1–3 years, and >3 years). Linear regression analysis was utilized to investigate the relationship between the LnRR of mass loss, carbon remaining, nitrogen remaining, lignin remaining, and geographic characteristics and initial litter traits, including latitude, longitude, MAT, MAP, soil respiration, initial carbon, nitrogen, lignin, and experimental duration. In addition, random-forest analysis was used to rank the importance of factors influencing the response of litter mass loss, which was determined using the percent increase in mean square error (%IncMSE) metric. Negative %IncMSE values indicate the lack of importance of predictors (Liaw and Wiener, 2002).

## 3 Results

### 3.1 Responses of litter decomposition to solar radiation in a global scale

**Figure 2:**
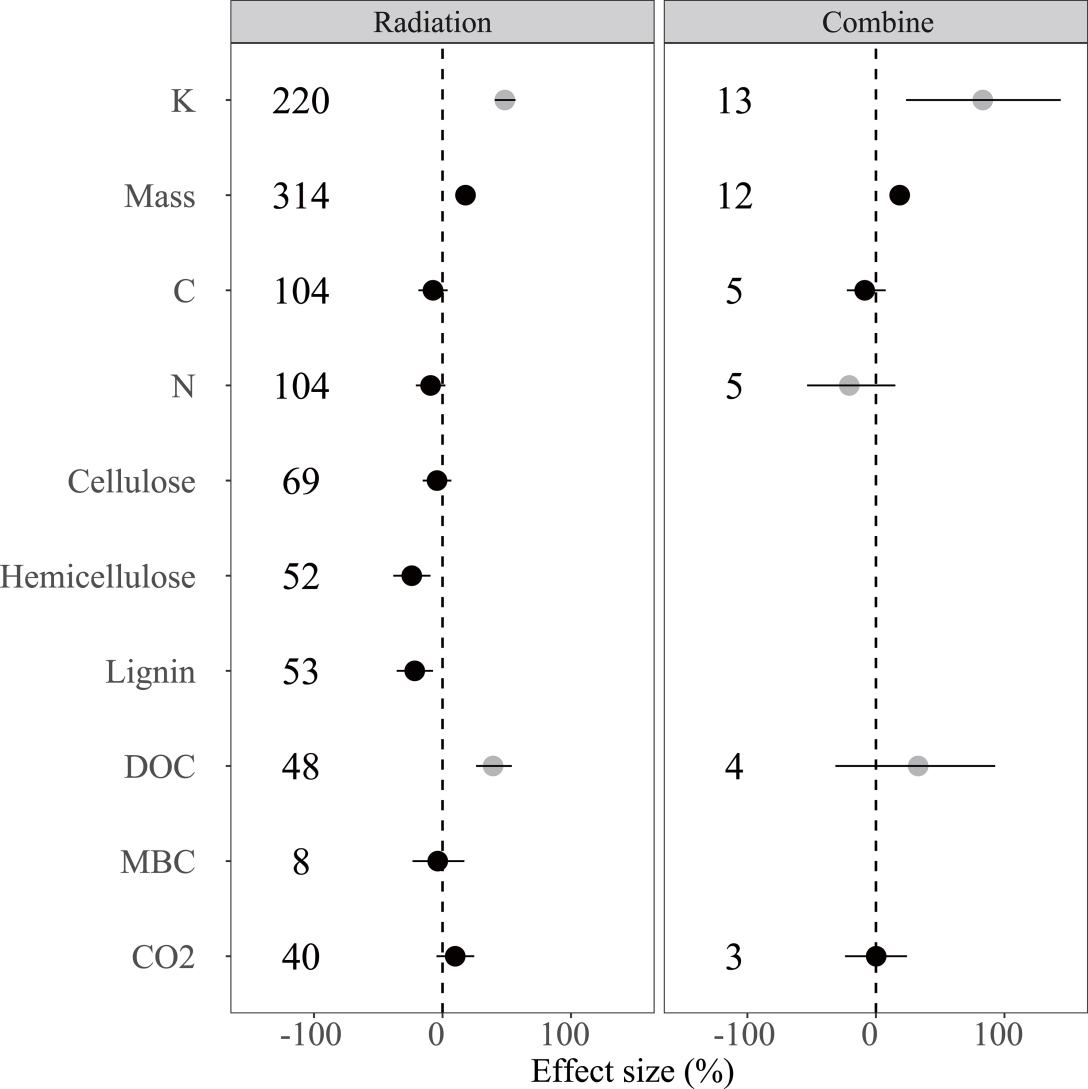
Responses of litter decomposition variables to solar radiation were examined through sole radiation treatment (Radiation) and the combination of water addition and radiation treatment (Combine). The results are presented as LnRR++ with 95%CI. The numbers indicate the number of data observations. Black points indicate a significant effect, whereas grey points indicate an insignificant effect. The indexes used are as follows: K = K value, Mass = mass loss, C = carbon remaining, N = nitrogen remaining, DOC = dissolved organic carbon concentration, Lignin = lignin remaining, MBC = microbial biomass carbon, and CO_2_ = CO_2_ emission from litter decomposition. Numbers indicate the number of data observations.

Solar radiation played a significant role in litter decomposition (**Figure 2**). The results of the sole solar radiation treatment revealed that solar radiation increased mass loss by 17.89%; reduced C remaining by 7.41%, N remaining by 9.28%, cellulose remaining by 4.24%, hemicellulose remaining by 23.92%, lignin remaining by 21.62%, and MBC by 3.70%; and increased CO_2_ emission by 9.99%. In the combined treatment, mass loss was increased by 18.05%, and C remaining was decreased by 8.73%, whereas CO_2_ emission was increased slightly by 0.20%. No significant difference in mass loss and C remaining was observed between the “Radiation” and “Combined” treatments. In addition, mass loss, C remaining, and N remaining had no significant difference among different experimental durations but had remarkable differences in different ecosystems and species, which closely correlated with precipitation in our database (**Figure S1**).

**Figure 3:**
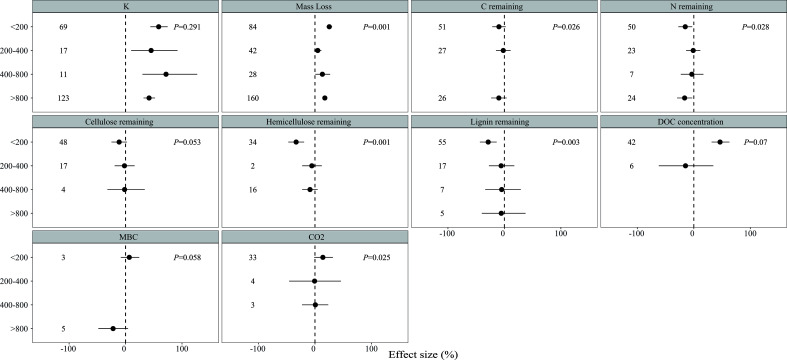
Effects of solar radiation on litter decomposition variables at different precipitation levels: <200mm, 200–400 mm, 400–800 mm, and >800 mm. Results were presented as LnRR++ with 95% confidence intervals. Numbers indicate the number of data observations. P indicates the result of the between-group heterogeneity test (QB) at different precipitation levels.

In assessing the influence of precipitation on the net effect of solar radiation, we verify whether the response of litter decomposition to solar radiation varies with precipitation amount. Significant variations in the effect of solar radiation on litter decomposition and CO_2_ emission were observed across different precipitation levels (**Figure 3**). Mass loss was significantly increased by 25.33% and 17.46% at precipitation levels below 200 mm and above 800 mm, whereas the increase was only 4.90% and 13.13% at precipitation levels of 200–400 mm and 400–800 mm, respectively. Similarly, the C remaining was reduced by 9.67% and 9.85% at precipitation levels below 200 mm and above 800 mm, respectively, and by 1.99% at a precipitation level of 200–400 mm. The N remaining was reduced by 15.17% and 16.30% at precipitation levels below 200 mm and above 800 mm but only 1.07% and 3.55% at precipitation levels of 200–400 mm and 400–800 mm, respectively. The reduction of the hemicellulose and lignin remaining was also greater at precipitation levels below 200 mm than that at other levels. Solar radiation significantly increased CO_2_ emission by 13.97%, but it only showed a slight effect at precipitation levels of 200–400 mm and 400–800 mm.

### 3.2 The relationships between the response ratio of mass loss and various factors

**Figure 4:**
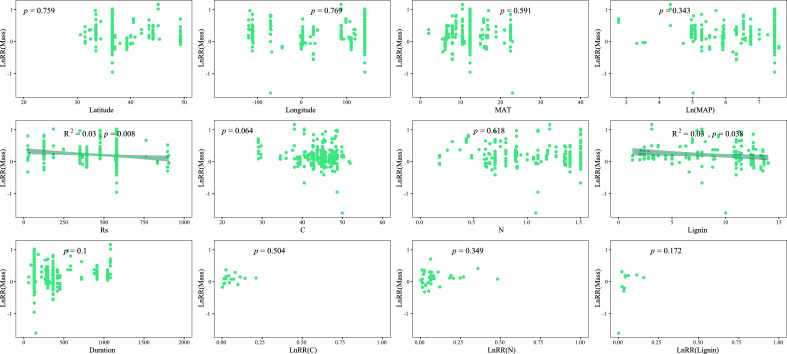
Relationships between the log response ratio (LnRR) of mass loss (Mass Loss) and various factors, including latitude, longitude, MAT, ln(MAP), soil respiration, initial litter C, N, lignin concentration, and experimental duration, as well as the log response ratios (LnRR) of the litter C and N remaining to solar radiation.

The results showed that the response of mass loss to solar radiation was only negatively related to soil respiration, initial lignin concentration, and ln(MAP) on a global scale (**Figure 4**). The response of C remaining showed a positive relationship with latitude, longitude, and initial lignin concentration and a negative relationship with MAT and experimental duration (**Figure S2**). The response of N remaining was positively related to latitude, ln(MAP), soil respiration, initial C, and initial lignin concentration and negatively related to MAT (**Figure S3**). The response of lignin remaining was positively related to ln(MAP), initial C, and initial lignin concentration and negatively related to initial N and experimental duration (**Figure S4**).

### 3.3 Dominant factors regulating the responses of mass loss to radiation

**Figure 5:**
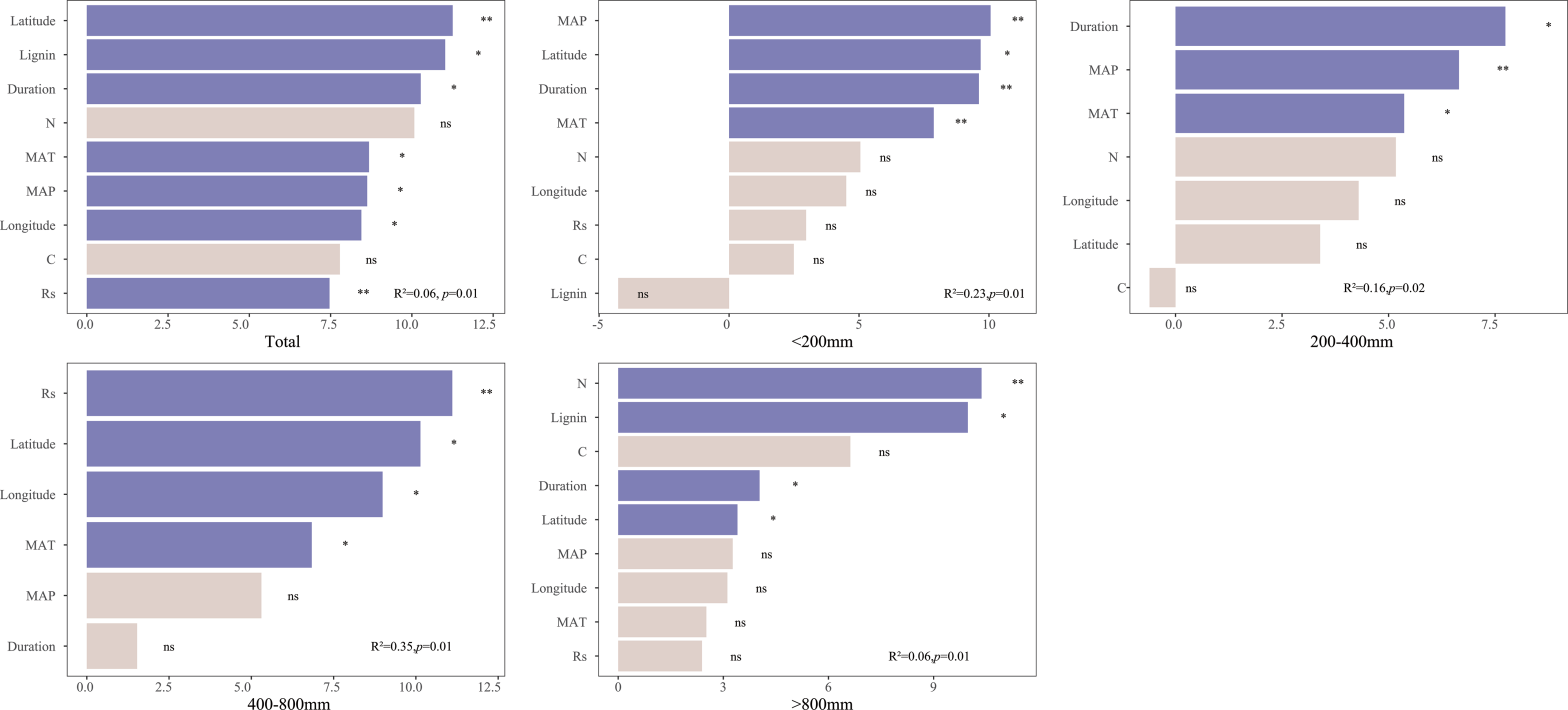
Random forest analysis was used to assess the importance of predictors of the log response ratio of mass loss to solar radiation on the global scale at precipitation levels of<200 mm, 200–400 mm, 400–800 mm, and >800 mm. The purple bar indicates the predictors that significantly influence mass loss, and the white bar indicates the predictors that exerted no significant influence on mass loss.

On a global scale, random-forest analysis revealed that several factors were significantly associated with the effects of solar radiation on mass loss, including latitude, initial lignin concentration, experimental duration, MAT, MAP, longitude, and soil respiration (**Figure 5**). When precipitation was less than 200 mm, MAP plays an important role in regulating the response of mass loss to radiation. However, as precipitation increased, the relative importance of MAP in regulating mass loss decreased with MAP exerting no significant effect on the response ratio when the precipitation level was between 400–800 mm and >800 mm. At a precipitation level of 400–800 mm, soil respiration plays an important role in regulating the ratio, and the initial litter trait such as initial N and lignin concentration became increasingly important in driving the response of mass loss to radiation when the precipitation level was >800 mm.

## 4 Discussion

### 4.1 Effects of the interactive effect on litter decomposition

Solar radiation is a critical factor in the regulation of litter decomposition. Our findings are consistent with previous research, demonstrating that solar radiation significantly increases mass loss in sole radiation and combined treatment. Moreover, the response of litter decomposition to solar radiation is closely related to MAP (**Figure 4**). In addition, no significant difference in the effect of solar radiation on mass loss is found among lands with precipitation levels below 200 mm and above 800 mm, which was higher than in other areas (**Figure 3**). In general, radiation plays a significant role in litter decomposition in extremely arid lands, (King et al., 2012; Austin et al., 2016; Berenstecher et al., 2020), whereas the contribution of photodegradation decreases in relatively humid ecosystems because of the increased microbes and plant canopy (Egidi et al., 2023; Smith et al., 2010; Evans et al., 2020). Contrary to previous studies indicating that radiation generally has a negligible effect on litter decomposition in wet ecosystems (Newsham et al., 1997; Moody et al., 2001), our results suggested that litter decomposition has a greater response to radiation at a precipitation level of >800 mm than those at 200–400 mm and 400–800 mm. These results answered questions (1) and (3), that is, the effect of radiation initially decreases and then increases with different precipitation levels, indicating that precipitation can change the litter decomposition mechanism. This result might be due to the condition of microbes in humid ecosystems where microbial biomass and diversity are significantly higher than in other areas, and previous research has shown that several soluble materials are produced by microbes, which exerted a positive effect on litter photodegradation (Gallo et al., 2006; Austin et al., 2009; Smith et al., 2010).

Classical models of global carbon turnover often fail to consider the impact of photodegradation on C loss during litter decomposition as previous studies have consistently neglected this factor. Our study suggests that sole radiation treatment and combined treatment had a significant but relatively minor effect on C remaining; however, a significant difference in precipitation amounts was observed (**Figure 3**). The response of C remaining to solar radiation was higher in areas with a precipitation level of<200 mm compared with other regions (except for a precipitation level of >800 mm). Similarly, the proportion of C, including cellulose, hemicellulose, and lignin, showed significant changes at a precipitation level of<200 mm, which supports our previous conclusion, that is, solar radiation has a greater impact in arid regions (Smith et al., 2010; Huang and Li, 2017). Solar radiation only decreased the lignin remaining at a precipitation level of<200 mm, and the mass loss and C, N, and lignin remaining had significant relationships with the initial litter lignin concentration (**Figures 4**, **S2**–**S4**), which is consistent with previous studies that have shown that lignin is the main target of photodegradation (Austin and Vivanco, 2006; King et al., 2012). Furthermore, solar radiation had a minor impact on lignin in other regions, particularly in areas where precipitation was greater than 800 mm, exhibiting similar mass loss and responses of C and N remaining to solar radiation in regions with a precipitation level of less than 200 mm (**Figure 3**). This finding indicated that lignin is more susceptible to solar radiation than to microbial decomposition (Vanderbilt et al., 2008; Bontti et al., 2009; Wang et al., 2015), which can directly absorb ultraviolet and short-wave visible light and produce smaller organic compounds (Austin and Vivanco, 2006; King et al., 2012). The relationship between the response of the lignin remaining and MAP also supports our view as the extent of the lignin remaining decreases with the increase of MAP (**Figure S4**). Therefore, increasing precipitation in the future will influence the mechanism driving lignin decomposition by altering the dominance of photodegradation and microbial decomposition. In addition, solar radiation increased litter DOC concentration at a precipitation level of<200 mm, although the effect was not significant. We can partly predict that the DOC concentration will increase with the increase in precipitation because of the breakdown and leaching effects (Smith et al., 2010; Austin, 2011; Huang and Li, 2017). This phenomenon will generate a positive feedback loop that improves litter decomposition decay because of its susceptibility to radiation and the provision of high-quality labile carbon to microbes.

Microbial activity primarily regulates the dynamics of litter N, with initial N continuously increasing because of microbial immobilization for growth, followed by a decrease as microbes mineralize N in later stages of decomposition (Brandt et al., 2007; Smith et al., 2010; Wang et al., 2015). In sole solar radiation treatment, N immobilization decreased (**Figure 2**), with a greater decline at a precipitation level of<200 mm (**Figure 3**). This trend is consistent with solar radiation exposure reducing microbial activity because biotic processes are inhibited (McLeod et al., 2008; Gliksman et al., 2017; Huang and Li, 2017; Marinho et al., 2020). However, the response of N remaining to solar radiation was not significant when precipitation was between 200-400 mm and 400-800 mm. These neutral results indicated that microbes are more resilient to the negative effects of radiation when moisture is not limited, and models of litter N dynamics should consider the mitigating effects of precipitation on microbial activity (King et al., 2012; Austin et al., 2016; Berenstecher et al., 2020). Compared with the moderately negative effect in areas with precipitation between 200–400 mm and 400–800 mm, the negative effect of radiation on N immobilization is stronger in humid ecosystems (precipitation >800 mm) where microbial decomposition dominantly regulates the process. In wet ecosystems, solar radiation can speed up nitrogen mineralization by microorganisms, thereby reducing the nitrogen remaining (Smith et al., 2010; Wei et al., 2022). Therefore, microbial decomposition is typically faster in areas with higher moisture content and more likely to progress to later stages of degradation (Austin and Vivanco, 2006; Smith et al., 2010; Gliksman et al., 2017). Thus, conducting long-term and short-term interactive N dynamic studies in different ecosystems is necessary to provide a comprehensive understanding of the interactive effects of multiple factors on N dynamics.

### 4.2 Effects of the interactive effect on microbial activity and CO_2_ emission

The inhibition of microorganisms by radiation may occur through several distinct mechanisms, such as DNA damage and decreased spore germination (Moody et al., 2001; Johnson, 2003; Austin and Vivanco, 2006; Parton et al., 2007; Austin, 2011), and modifications to the structure of microbial communities (Pancotto et al., 2003; Smith et al., 2010; Sala et al., 2015; Marinho et al., 2020). Although the precise role of water in this process remains unclear, microorganisms are more resilient to harm when not experiencing water stress. However, our meta-analysis found that solar radiation had a negative impact on MBC, particularly in ecosystems with high precipitation (>800 mm), whereas MBC increased in ecosystems with low precipitation (<200 mm) probably because the positive effect of photodegradation on microbial activity offsets the negative effect in arid lands. As the moisture level increases, the primary effect of radiation on litter decomposition may transition from “photopriming” to the suppression of microorganisms. However, given the restricted availability of MBC data, further experiments must be conducted to confirm this conclusion. Therefore, conducting experiments in the future is necessary to examine the interaction between water and radiation on MBC and elucidate the changes in the mechanism of litter decomposition under increasing precipitation. In addition, although the change in MBC was unclear, CO_2_ emission increased across all treatments and precipitation levels (except 200-400 mm). This increase was due to the emission of C-based trace gases produced by the photochemical mineralization of recalcitrant compounds (McLeod et al., 2008; Rutledge et al., 2010). However, the response of CO_2_ emission to solar radiation decreased with the increase of precipitation compared with the response when the precipitation level was<200 mm. This finding provides a new insight into the influence of precipitation on CO_2_ emission, where CO_2_ emission may significantly change with the increase of precipitation in photodegradation ways, particularly in xeric lands. However, given the data limitations, CO_2_ obtained from global photomineralized sources cannot be quantified. Thus, future research projects are necessary to gain a more integrated understanding of the effects of radiation on CO_2_ emission.

### 4.3 Dominant factors regulating the responses of the mass loss to solar radiation

The impact of solar radiation on litter decomposition is complex, and it varies depending on its magnitude and other factors. On a global scale, latitude and initial lignin concentration play crucial roles in regulating the response ratio of mass loss to solar radiation. This finding supports earlier studies that consider lignin as the primary target for photodegradation (Austin and Vivanco, 2006; Brandt et al., 2007; Wang et al., 2022). Similarly, initial litter traits, such as N and lignin concentration, play important roles in regulating the response ratio of mass loss to solar radiation at a precipitation level of >800 mm, which might be due to no moisture limitation in this region; moreover, intense microbial activity was observed, and the photodegradation activity was not limited by other factors (Coûteaux et al., 1995). However, climatic factors appear to have a greater influence on litter decomposition than initial litter traits in ecosystems that are moisture limited, which answers our second question (2) and presents challenges in predicting global litter decomposition. With the expected increase in precipitation in the future, MAP may become more significant and affect CO_2_ emission through leaching and microbial growth. Therefore, long-term experiments that consider biotic and abiotic factors, including MAP, are essential for predicting litter decomposition and CO_2_ emission in a changing climate.

## 5 Conclusion and prospects

We conducted a meta-analysis and found that the effect of radiation on litter decomposition depends on the magnitude of MAP. In particular, a similar effect of radiation on litter mass loss was observed in arid lands and humid ecosystems, and precipitation can increase resilience to the negative effects of radiation on microbes. Furthermore, the mechanism of litter decomposition and the regulatory factors of photodegradation would change with increasing precipitation. Moreover, radiation increased the CO_2_ emission rate for all precipitation amounts, particularly in arid lands. As precipitation continues to increase in the future, litter decomposition, carbon cycling, and CO_2_ emission caused by photodegradation may change significantly.

We also found that the heterogeneity of radiation’s impact on litter decomposition can be explained by climatic factors and initial litter traits. This knowledge may help us comprehensively understand the mechanism by which radiation affects litter decomposition and CO_2_ emission in different ecosystems. Future studies should focus on investigating the interactive effect of precipitation and solar radiation on litter decomposition and microbial activity in relatively humid ecosystems to obtain a comprehensive understanding of how climate change will affect litter decomposition. Furthermore, more research must be conducted to accurately assess the effect of radiation on CO_2_ emission caused by photodegradation. A comprehensive understanding of the contribution of biodegradation and photodegradation to litter decomposition will help us in predicting how litter decomposition will respond to climate change.

## Data availability statement

The original contributions presented in the study are included in the article/**Supplementary Material**. Further inquiries can be directed to the corresponding author.

## Author contributions

YL analyzed data and wrote the manuscript. LL and XL conceived the work and supervised the research. SW contributed to analyze the manuscript. All authors contributed to the article and approved the submitted version.
