# Supplementary material for: Precipitation modulates the net effect of solar radiation on litter decomposition and CO2 emission - a meta-analysis
Source: Front Plant Sci. 2023 Jul 5;14:1200155. doi: 10.3389/fpls.2023.1200155 (PMC10356545; doi:10.3389/fpls.2023.1200155)
Supplement: Supplementary file 1 [file DataSheet_1.docx]

**TABLE S1 |Studies included in this meta-analysis**

Almagro, M., Martínez-López, J., Maestre, F.T., Bowker, M.A., & García-Palacios, P. (2017). The contribution of photodegradation to litter decomposition in semiarid Mediterranean grasslands depends on its interaction with local humidity conditions, litter quality and position. Ecosystems, 20, 527–542.

Austin, A.T., & Vivanco, L. (2006). Plant litter decomposition in a semi-arid ecosystem controlled by photodegradation. Nature, 442, 555–558.

Baker, N.R., & Allison, S.D. (2015). Ultraviolet photodegradation facilitates microbial litter decomposition in a Mediterranean climate. Ecology, 96(7), 1994-2003.

Barnes, P.W., Throop, H.L., Hewins, D.B., & Abbott, L.K. (2012). Soil coverage reduces photodegradation and promotes the development of soil-microbial films on dryland leaf litter. Ecosystems, 15, 311–321.

Brandt, L.A., King, J.Y., & Milchunas, D.G. (2007). Effects of ultraviolet radiation on litter decomposition depend on precipitation and litter chemistry in a shortgrass steppe ecosystem. Global Change Biology, 13, 2193-2205.

Brandt, L.A., King, J.Y., Hobbie, S.E., & Milchunas, D.G. (2010). The role of photodegradation in surface litter decomposition across a grassland ecosystem precipitation gradient. Ecosystems, 13, 765–781.

Day, T.A., Urbine, J.M., & Bliss, M.S. (2022). Supplemental precipitation accelerates decay but only in photodegraded litter and implications that sunlight promotes leaching loss. Biogeochemistry, 158, 113–129.

Day, T.A., Bliss, M.S., Tomes, A.R., Ruhland, C.T., & Guénon, R. (2018). Desert leaf litter decay: Coupling of microbial respiration, water-soluble fractions and photodegradation. Global Change Biology, 24, 5454–5470.

Erdenebileg, E., Ye, X., Wang, C., Huang, Z., Liu, G., & Cornelissen, J. H. C. (2018). Positive and negative effects of UV irradiance explain interaction of litter position and UV exposure on litter decomposition and nutrient dynamics in a semi-arid dune ecosystem. Soil Biology and Biochemistry, 124, 245-254.

Huang, G., Zhao, H.-M., & Li, Y. (2017). Litter decomposition in hyper-arid deserts: Photodegradation is still important. Science of The Total Environment, 601–602, 784-792.

Huang, G., & Li, Y. (2017). Photodegradation effects are related to precipitation amount, precipitation frequency and litter traits in a desert ecosystem. Soil Biology and Biochemistry, 115, 383-392.

Gehrke, C., Björn, L. O., Callaghan, T. V., & Sonesson, M. (1995). The impact of enhanced ultraviolet-B radiation on litter quality and decomposition processes in Vaccinium leaves from the Subarctic. Oikos, 72, 213-222.

Gliksman, D., Rey, A., Seligmann, R., Dumbur, R., Sperling, O., Navon, Y., Haenel, S., De Angelis, P., Arnone, J. A., III, & Grünzweig, J. M. (2017). Biotic degradation at night, abiotic degradation at day: Positive feedbacks on litter decomposition in drylands. Global Change Biology, 23, 1564-1574.

Jiang, H., Pan, Y., Liang, J., Yang, Y., Chen, Q., Lv, M., Pang, L., He, W., & Tian, X. (2022). UV radiation doubles microbial degradation of standing litter in a subtropical forest. Journal of Ecology, 110, 2156–2166.

Predick, K. I., Archer, S. R., Aguillon, S. M., Keller, D. A., Throop, H. L., & Barnes, P. W. (2018). UV-B radiation and shrub canopy effects on surface litter decomposition in a shrub-invaded dry grassland. Journal of Arid Environments, 157, 13-21.

Lin, Y., & King, J. Y. (2014). Effects of UV exposure and litter position on decomposition in a California grassland. Ecosystems, 17, 158-168.

Lin, Y., Scarlett, R. D., & King, J. Y. (2015). Effects of UV photodegradation on subsequent microbial decomposition of Bromus diandrus litter. Plant and Soil, 395, 263-271.

Gallo, M. E., Porras-Alfaro, A., Odenbach, K. J., & Sinsabaugh, R. L. (2009). Photoacceleration of plant litter decomposition in an arid environment. Soil Biology and Biochemistry, 41(7), 1433-1441.

Méndez, M. S., Martinez, M. L., Araujo, P. I., et al. (2019). Solar radiation exposure accelerates decomposition and biotic activity in surface litter but not soil in a semiarid woodland ecosystem in Patagonia, Argentina. Plant and Soil, 445, 483-496.

Moody, S. A., Paul, N. D., Björn, L. O., et al. (2001). The direct effects of UV-B radiation on Betula pubescens litter decomposing at four European field sites. Plant Ecology, 154, 27-36.

Newsham, K. K., McLeod, A., Roberts, J. D., Greenslade, P. D., Emmett, B. A. (1997). Direct effects of elevated UV-B radiation on the decomposition of Quercus robur leaf litter. Oikos, 79(3), 592-602.

Marinho, O. A., Martinelli, L. A., Duarte-Neto, P. J., Mazzi, E. A., & King, J. Y. (2020). Photodegradation influences litter decomposition rate in a humid tropical ecosystem, Brazil. Soil Biology and Biochemistry, 150, 107979.

Pancotto, V. A., Sala, O. E., Cabello, M., López, N. I., Robson, T. M., Ballaré, C. L., Caldwell, M. M., & Scopel, A. L. (2003). Solar UV-B decreases decomposition in herbaceous plant litter in Tierra del Fuego, Argentina: potential role of an altered decomposer community. Global Change Biology, 9, 1465-1474.

Pancotto, V. A., Sala, O. E., Robson, T. M., Caldwell, M. M., & Scopel, A. L. (2005). Direct and indirect effects of solar ultraviolet-B radiation on long-term decomposition. Global Change Biology, 11, 1982-1989.

Berenstecher, P., Vivanco, L., Pérez, L. I., Ballaré, C. L., & Austin, A. T. (2020). Sunlight doubles aboveground carbon loss in a seasonally dry woodland in Patagonia. Current Biology, 30(16), R976-R977.

Pieristè, M., Neimane, S., Solanki, T., Nybakken, L., Jones, A. G., Forey, E., Chauvat, M., Ņečajeva, J., & Robson, T. M. (2020). Ultraviolet radiation accelerates photodegradation under controlled conditions but slows the decomposition of senescent leaves from forest stands in southern Finland. Plant Physiology and Biochemistry, 146, 42-54.

Pieristè, M., Chauvat, M., Kotilainen, T. K., Liski, J., Munson, A. D., Nybakken, L., Ņečajeva, J., & Robson, T. M. (2019). Solar UV-A radiation and blue light enhance tree leaf litter decomposition in a temperate forest. Oecologia, 191, 191-203

Rozema, J., Tosserams, M., Nelissen, H. J. M., Broekman, R. A., van de Staaij, J. W. M., & van de Geijn, S. C. (1997). Stratospheric ozone reduction and ecosystem processes: Enhanced UV-B radiation affects chemical quality and decomposition of leaves of the dune grassland species Calamagrostis epigeios. Plant Ecology, 128, 285-294.

Yanni, S. F., Suddick, E. C., & Six, J. (2015). Photodegradation effects on CO2 emissions from litter and SOM and photo-facilitation of microbial decomposition in a California grassland. Soil Biology and Biochemistry, 91, 40-49. https://doi.org/10.1016/j.soilbio.2015.08.021

Smith, W. K., Gao, W., Steltzer, H., Wallenstein, M. D., & Tree, R. (2010). Moisture availability influences the effect of ultraviolet-B radiation on leaf litter decomposition. Global Change Biology, 16, 484-495.

Song, X. Z., Zhang, H. L., Chang, S. X., et al. (2012). Elevated UV-B radiation increased the decomposition of Cinnamomum camphora and Cyclobalanopsis glauca leaf litter in subtropical China. Journal of Soils and Sediments, 12, 307-311.

Day, T. A., Guénon, R., & Ruhland, C. T. (2015). Photodegradation of plant litter in the Sonoran Desert varies by litter type and age. Soil Biology and Biochemistry, 89, 109-122.

Wang, Q. W., Pieristè, M., Liu, C., Kenta, T., Robson, T. M., & Kurokawa, H. (2021). The contribution of photodegradation to litter decomposition in a temperate forest gap and understorey. New Phytologist, 229(5), 2625-2636.

Wang, J., Yang, S., Zhang, B., et al. (2017). Temporal dynamics of ultraviolet radiation impacts on litter decomposition in a semi-arid ecosystem. Plant and Soil, 419, 71-81.

Song, X., Jiang, H., Zhang, Z., Zhou, G., Zhang, S., & Peng, C. (2014). Interactive effects of elevated UV-B radiation and N deposition on decomposition of Moso bamboo litter. Soil Biology and Biochemistry, 69, 11-16.

Lin, Y., Karlen, S. D., Ralph, J., & King, J. Y. (2018). Short-term facilitation of microbial litter decomposition by ultraviolet radiation. Science of the Total Environment, 615, 838-848.

**TABLE S2 |** Between-group heterogeneity test (*Q*B) for different precipitation levels

| Variables | Qb | P |
| --- | --- | --- |
| K | 5.9116 | 0.291 |
| Mass | 248.9215 | 0.001 |
| C | 23.6507 | 0.026 |
| N | 10.4466 | 0.028 |
| Cellulose | 6.8965 | 0.053 |
| Hemicellulose | 58.6963 | 0.001 |
| Lignin | 24.3007 | 0.003 |
| DOC | 8.5357 | 0.07 |
| MBC | 8.7354 | 0.058 |
| CO2 | 10.7716 | 0.025 |

**TABLE S3 |** Between-group heterogeneity test (*Q*B) for different ecosystem，experimental duration, and species

| Variables | Ecosystem |  | Duration |  | Species |  |
| --- | --- | --- | --- | --- | --- | --- |
|  | Qb | P | Qb | P | Qb | P |
| Mass | 37.2224 | 0.001 | 9.7201 | 0.071 | 11.462 | 0.047 |
| C | 7.8053 | 0.25 | 164.5036 | 0.001 | 4.0629 | 0.512 |
| N | 65.5783 | 0.006 | 199.4892 | 0.001 | 1.7693 | 0.656 |


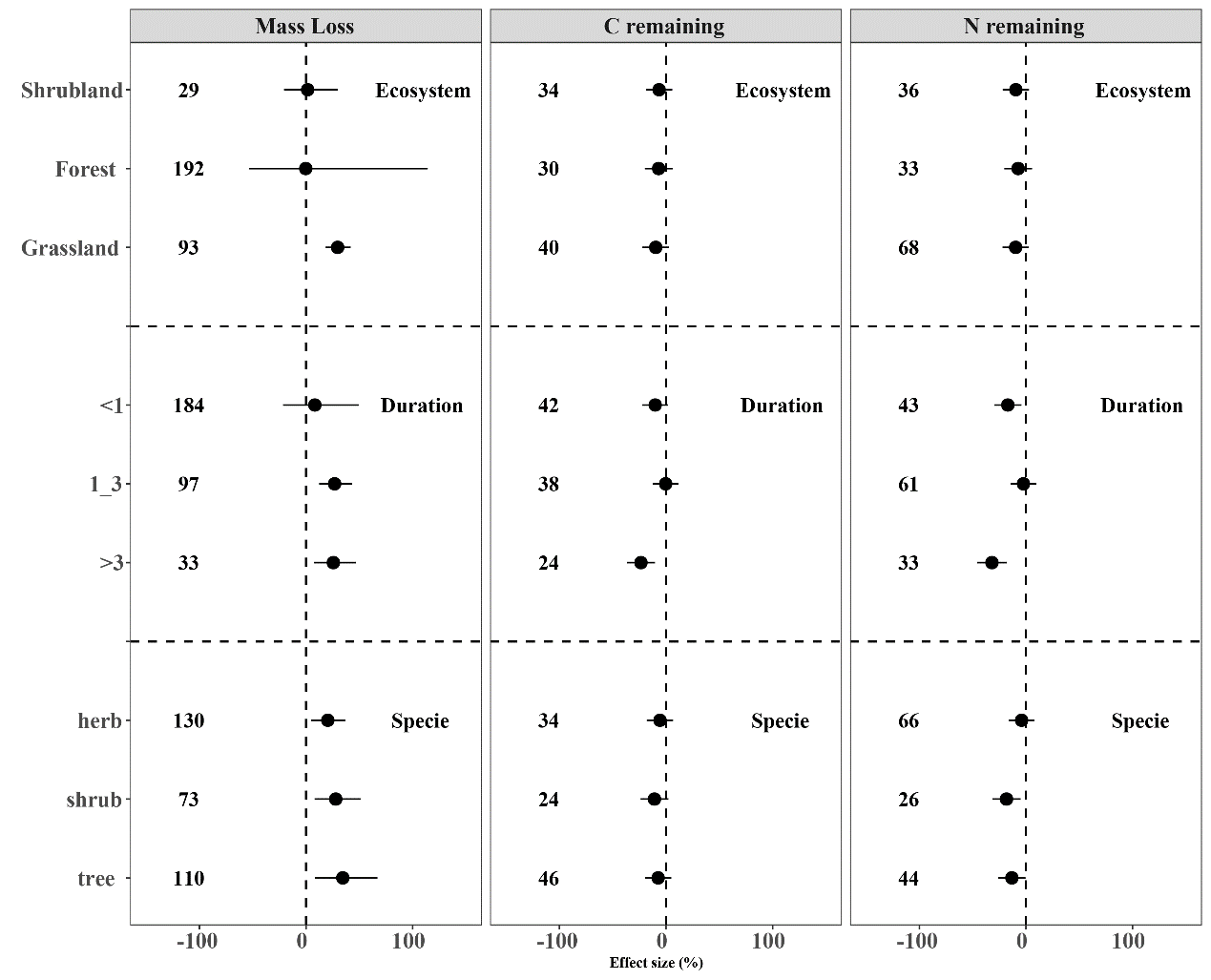


Figure S1 Responses of litter decomposition variables to solar radiation for different ecosystem includes “Shrubland”,“Forest”,“Grassland”; different experimental years includes: “<1”, “1-3”, and “>3” years; different species includes: “grass”,“shrub”, and “tree”. Values are LnRR and 95%CI. Numbers in parenthesis indicate the number of data observations, numbers outside of parenthesis indicate the number of studies including the indexes.


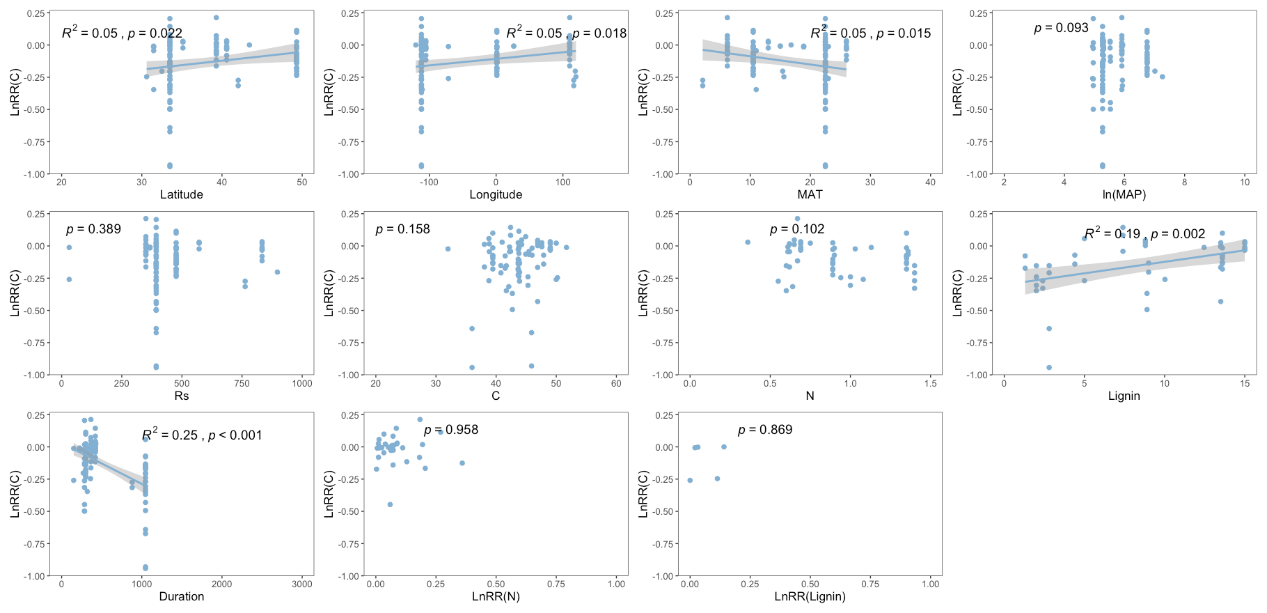


Figure S2 Relationships between the log response ratio (LnRR) of C remaining and latitude, longitude, MAT, ln(MAP), soil respiration, initial litter C, N, lignin concentration, experimental duration, and log response ratios (LnRR) of litter N remaining and lignin remaining to radiation.


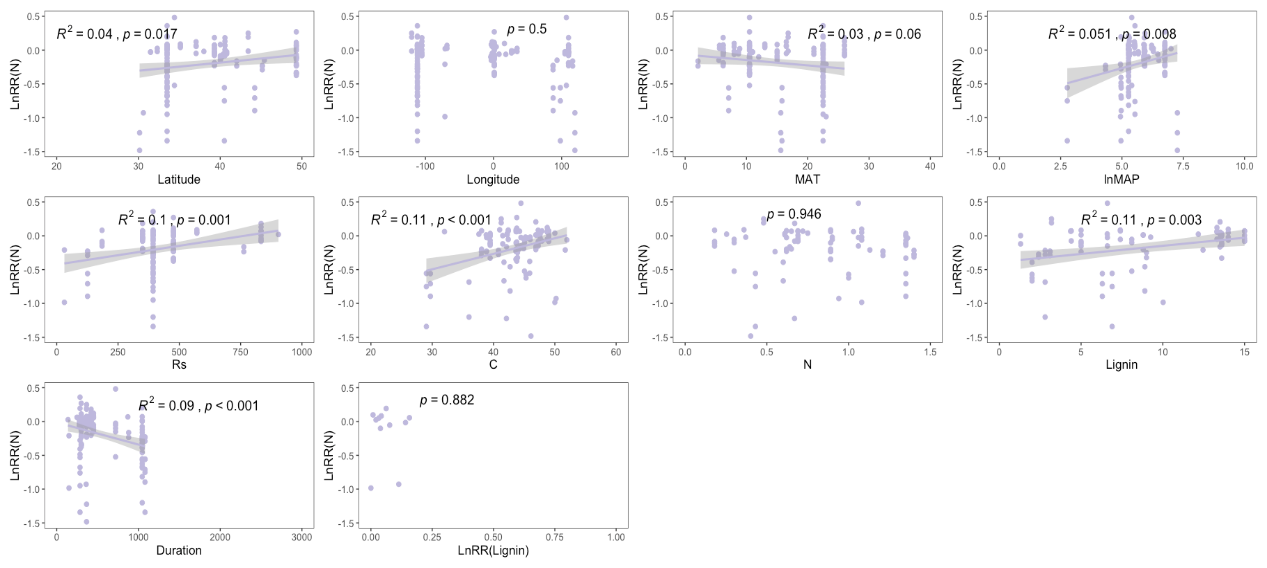


Figure S3 Relationships between the log response ratio (LnRR) of N remaining and latitude, longitude, MAT, ln(MAP), soil respiration, initial litter C, N, lignin concentration, experimental duration, and log response ratios (LnRR) of litter lignin remaining to radiation.


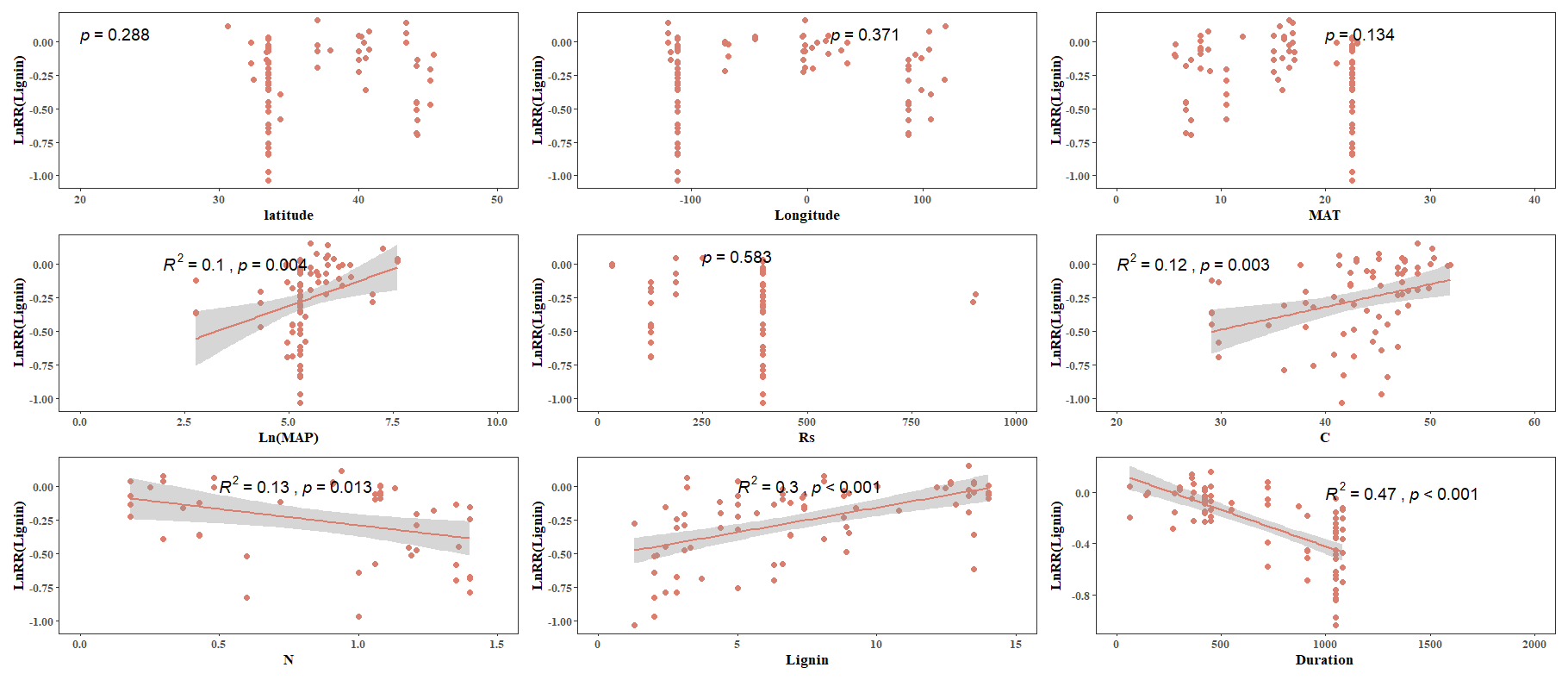


Figure S4 Relationships between the log response ratio (LnRR) of Lignin remaining and latitude, longitude, MAT, ln(MAP), soil respiration, initial litter C, N, lignin concentration, experimental duration to solar radiation.
